# Acoustic localization at large scales: a promising method for grey wolf monitoring

**DOI:** 10.1186/s12983-018-0260-2

**Published:** 2018-04-12

**Authors:** Morgane Papin, Julian Pichenot, François Guérold, Estelle Germain

**Affiliations:** 1 Centre de Recherche et d’Observation sur les Carnivores (CROC), 4 rue de la banie, 57590, Lucy, France; 2 Université de Lorraine, CNRS, Laboratoire Interdisciplinaire des Environnements Continentaux, F-57500 Metz, France; 3 Biologiste Écologue Consultant (BEC), 8A rue principale, 57590, Fonteny, France

**Keywords:** acoustic monitoring, autonomous recorders, *Canis lupus*, field research, localization estimation, microphone array, wolf howl

## Abstract

**Background:**

The grey wolf (*Canis lupus*) is naturally recolonizing its former habitats in Europe where it was extirpated during the previous two centuries. The management of this protected species is often controversial and its monitoring is a challenge for conservation purposes. However, this elusive carnivore can disperse over long distances in various natural contexts, making its monitoring difficult. Moreover, methods used for collecting signs of presence are usually time-consuming and/or costly. Currently, new acoustic recording tools are contributing to the development of passive acoustic methods as alternative approaches for detecting, monitoring, or identifying species that produce sounds in nature, such as the grey wolf. In the present study, we conducted field experiments to investigate the possibility of using a low-density microphone array to localize wolves at a large scale in two contrasting natural environments in north-eastern France. For scientific and social reasons, the experiments were based on a synthetic sound with similar acoustic properties to howls. This sound was broadcast at several sites. Then, localization estimates and the accuracy were calculated. Finally, linear mixed-effects models were used to identify the factors that influenced the localization accuracy.

**Results:**

Among 354 nocturnal broadcasts in total, 269 were recorded by at least one autonomous recorder, thereby demonstrating the potential of this tool. Besides, 59 broadcasts were recorded by at least four microphones and used for acoustic localization. The broadcast sites were localized with an overall mean accuracy of 315 ± 617 (standard deviation) m. After setting a threshold for the temporal error value associated with the estimated coordinates, some unreliable values were excluded and the mean accuracy decreased to 167 ± 308 m. The number of broadcasts recorded was higher in the lowland environment, but the localization accuracy was similar in both environments, although it varied significantly among different nights in each study area.

**Conclusions:**

Our results confirm the potential of using acoustic methods to localize wolves with high accuracy, in different natural environments and at large spatial scales. Passive acoustic methods are suitable for monitoring the dynamics of grey wolf recolonization and so, will contribute to enhance conservation and management plans.

## Background

Passive acoustic monitoring is being used increasingly to study species that produce sounds in their natural environments (e.g. vocalizations and stridulations) [1]. The current protocols based on passive acoustics methods allow the study of elusive and/or nocturnal species that live in harsh environments (e.g. dangerous access, thick vegetation or limited visibility) [2–4]. These protocols are focused on species detection [5], density estimation [6, 7], territory use [8], and localization [9, 10]. They are not technically limited to a time period, non-invasive and so, avoid interference with animal behavior in contrast to other monitoring methods (e.g. direct capture or the intrusive presence of observers in the field) [2, 11]. Passive acoustics may also help to reduce the time and human resources required in the field [12, 13]. These main features of passive acoustics suggest that this interesting approach could be employed for monitoring elusive species that require conservation or management plans, such as the grey wolf (*Canis lupus*).

During the two last centuries, the grey wolf was extirpated in many areas throughout Europe and North America [14]. In Europe, the species is now legally protected by the Bern Convention (1979) and the Habitats Directive (1992). As a consequence, wolves have been recolonizing their former areas in recent decades [14, 15]. However, conflicts emerge with humans where their ranges overlap with human settlement and agriculture mainly due to the predation on livestock [16, 17]. Thus, understanding and monitoring the expansion of the grey wolf’s range is important for preventing or mitigating conflicts as well as for conservation and management purposes. However, the monitoring of wolves is still challenging in the field because it is a wide-ranging habitat generalist, which lives at low densities and is often secretive and elusive [18, 19]. Moreover, the conventional methods used for detecting the presence of grey wolves and estimating their number and population dynamics can be very time-consuming and costly.

Studying howls may be a powerful approach for monitoring grey wolf populations, especially in the summer and during the mating season when howls are produced widely [20–22]. For instance, wolf howls can allow scientific and wildlife managers to identify a pack due to their acoustic structure [23, 24]. In addition, several studies performed in captivity have shown that wolf has individual vocal signature [25–28]. Other studies have highlighted the potential use of bioacoustics for detecting wolves [29] as well as for counting them [28, 30–33] or detecting reproduction events [13]. The results of these studies support the possibility of using acoustics for monitoring wolves in the wild. However, to our knowledge, very few studies have employed passive acoustics for monitoring wolves (e.g. [29]) and none for localizing them.

In the present study, we conducted field experiments to investigate the possibility of using a low-density microphone array to localize wolves at a large scale in two areas located in the colonization front of the species in north-eastern France [34–37]. For scientific and social reasons, the experiments were based on a synthetic sound with similar acoustic properties to wolf howls. As these areas were characterized by two contrasting environmental contexts (mid-mountain and lowland), the synthetic sound was broadcast at several sites defined according to a stratified sampling technique based on topography and land-use. We calculated localization estimates and the accuracy. Finally, we identified the parameters and biases that influenced the localization accuracy.

## Methods

### Study areas

**Fig. 1:**
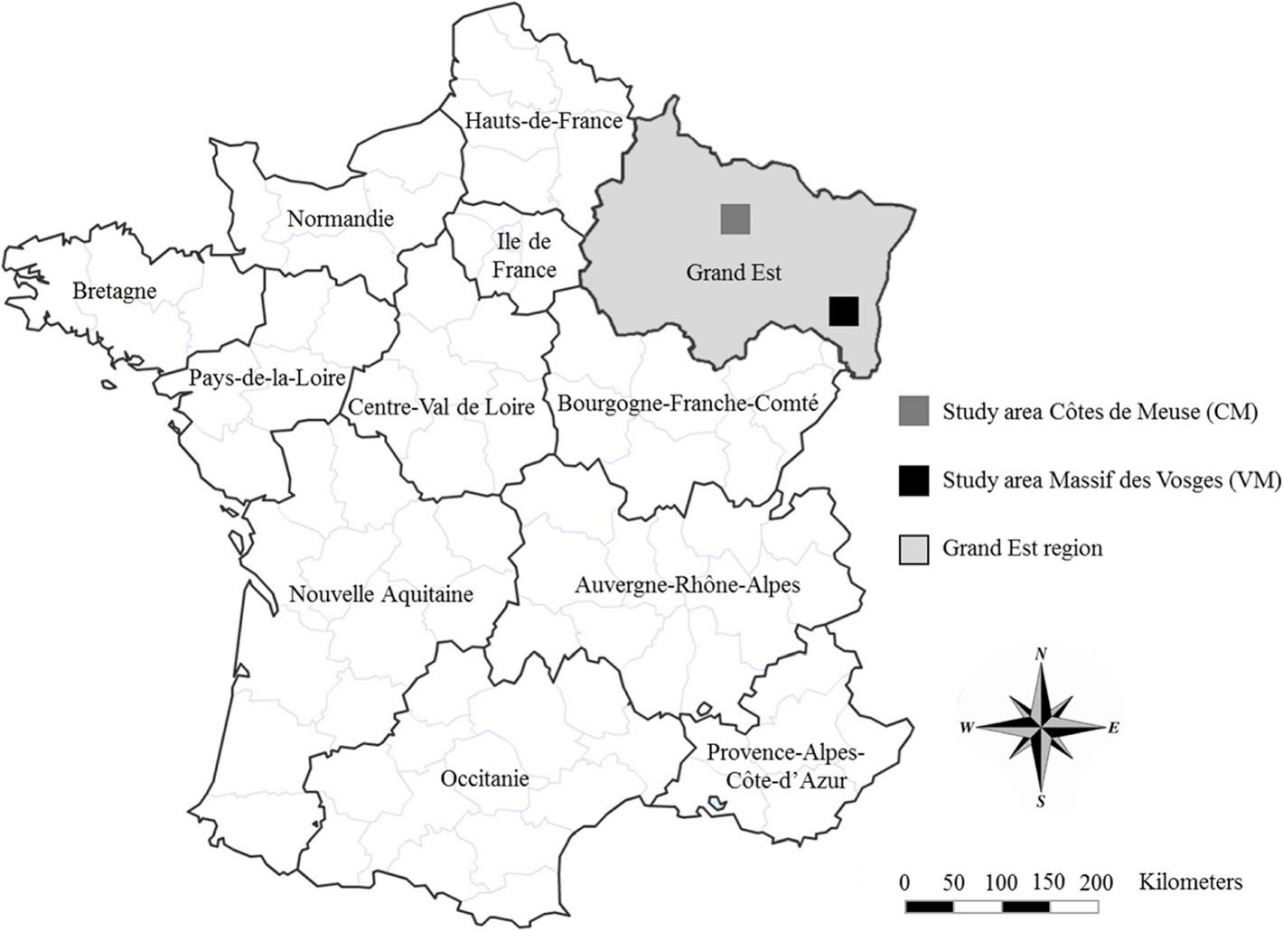
Locations of the two study areas in north-eastern France

The study was conducted in two different areas located in the colonization front of grey wolf in north-eastern France (Fig. 1). The first study area was located in a mid-mountain environment in the Massif des Vosges (VM), where the presence of a wolf pack (at least two individuals) has been attested since 2011 [34, 35, 37]. This area is covered by mainly herbaceous vegetation (22%), shrub (51%), and coniferous forest (27%), and the altitude ranges from 518 to 1305 m above sea level (mean: 930 m).

The second study area was located in the Côtes de Meuse (CM) at altitude ranging from 247 to 381 m above sea level (mean: 329 m), where the presence of the grey wolf was observed in 2012 [36]. The area is covered mainly by deciduous forest (90%) and open land with herbaceous vegetation accounts for only 10% of the area.

The grey wolf howls throughout the year but the periods with the most frequent howling activity are the breeding season (January to April: [20]) and the months following the birth of pups (August to October: [21]). Thus, this study was conducted during August 2015 in VM and August 2016 in CM. These periods also coincided with good conditions for access to the study areas.

### Sampling methods and microphone arrays

**Fig. 2:**
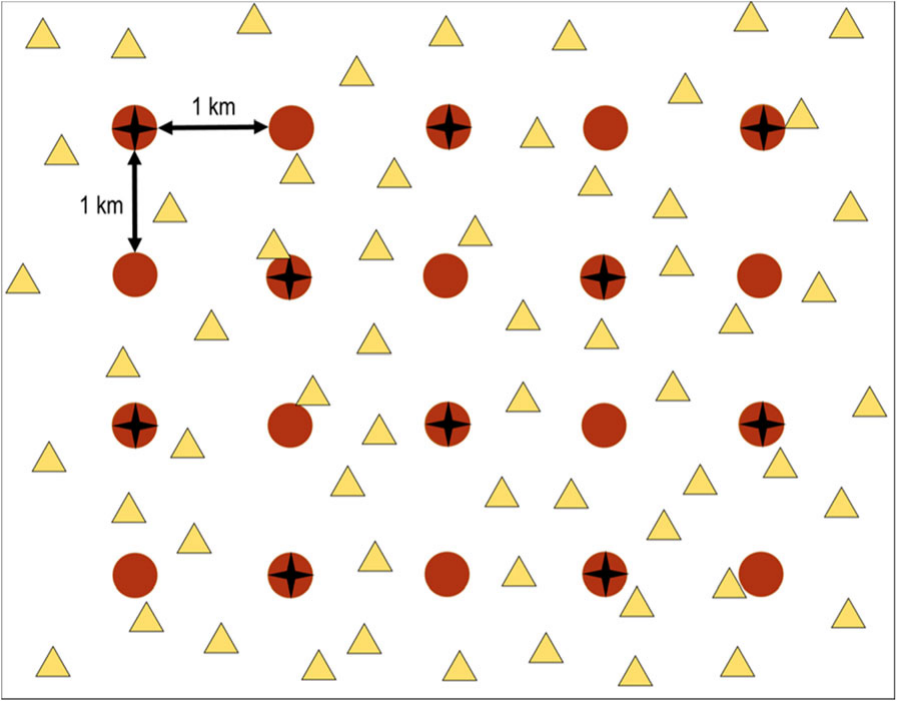
Sampling method employed in each study area. Red circles: autonomous recorders (20). Black stars: dataloggers (10). Yellow triangles: broadcast sites (60)

Twenty autonomous recorders were placed on a systematic grid with an area of 30 km^2^ (6 × 5 km; Fig. 2) at a regular spacing of 1 km, conducing to a relatively low recorder density (0.67 recorders per km^2^) for both study areas. The automatic recording units employed were Wildlife Acoustics Song Meters (model: SM3; Wildlife Acoustics Inc., Concord, MA, USA) with two built-in omnidirectional microphones (SM3-A1, bandpass: 20–20,000 Hz, frequency response: 20–20,000 Hz ± 10 dB). All of the recorders were associated with a global positioning system (GPS) unit (Garmin International Inc., Olathe, KS, USA) to synchronize their clock time automatically with high precision. The recorders collected 40 acoustic information channels in stereo using 16-bit .wav files at a sampling rate of 16,000 Hz. The recorders were programmed to operate from 8:55 PM to 8:54 AM and to generate 59-min files separated by a break of 1 min (ensure time synchronization). The gain was set to 24 dB for each channel.

The recorders were fixed to tree trunks at a height of 2.88 ± 0.49 m (mean ± standard deviation [SD]). Their locations were measured with a Trimble GPS (model: Juno 5B EGPS, real-time accuracy: 2–4 m; Trimble Navigation Limited, Sunnyvale, CA, USA).

### Broadcast sites and periods

In each study area, 60 broadcast sites were randomly distributed by stratified sampling according to the topography using the “*Topographic Position Index*” [38–40] and land-use using the “*Corine Land Cover*” code (European Union – SoeS, Corine Land Cover 2006) with QGIS software (version 2.8.1: [41]). The sites located away from roads were then moved to the closest road to allow access with a vehicle. The spatial sampling of the broadcast sites in terms of the distances to the autonomous recorders was similar in the two study areas (z-test: z = − 1.8180, α = 0.05).

The sound was broadcast during three consecutive nights from 9 PM to 6 AM, where each night was divided into three periods (dusk: 9 PM to 12 AM; night: 12 AM to 3 AM; dawn: 3 AM to 6 AM). For each night, a different itinerary was used so that each broadcast site was visited once during the three different periods. All of the broadcast sites locations were measured using the Trimble GPS.

### Synthetic sound and broadcast equipment

As the study of large carnivores is a sensitive subject [17, 42], we chose to use a synthetic sound with similar acoustic properties to wolf howls rather than using real howls. This sound also permitted to exclude the effects of wolves’ individual acoustic characteristics [25–28, 43]. It was created with the Seewave package [44] in R software (version 3.1.2). The sound comprised mixed pure tones of 7 s with fundamental frequencies ranging from 300 to 1000 Hz, which was accompanied by four harmonics that covered a wide range of the frequencies that can be found in wolf howls [20, 28, 45].

The sound was broadcast from four directional loudspeakers (model: MSH 30/BT, bandpass: 90–20,000 Hz, output: 50 W at 8 Ω; Work Pro CA, Valencia, Spain) connected to a mixing amplifier (model: PA 90/2 USB, frequency response: 80–18,000 Hz ± 3 dB; output: 30 W RMS; Work Pro CA) and a 12 V battery. The loudspeakers were attached to a car roof. During each broadcast, a digital sound level meter was employed to control the intensity level at 1 m (model: FI 70SD, bandpass: 31.5–8000 Hz, frequency response: 8000 Hz ± 5.6 dB, settings: fast response, A-weighting; Distrame S.A, Sainte-Savine, France).

### Meteorological context

The nights were selected according to the optimal meteorological conditions for acoustic experimentation, i.e. very low wind speed and no rainfall. The wind speed was measured for 1 min at each broadcast site with an anemometer (model: WS9500; La Crosse Technology, Geispolsheim, France) and it was always less than 2 m.s^− 1^. In addition, 10 weather dataloggers (model: DT-174B; Center for Educational Measurement Inc., Makati, Philippines) were installed below 10 recorders to record the air temperature every 2 min (Fig. 2). The temperature data acquired by all of the dataloggers were averaged per night period and per night. They were used subsequently to calculate the speed of sound during the nocturnal broadcast period, which was required for localization estimation.

### Analysis of recordings and localization estimates

**Fig. 3:**
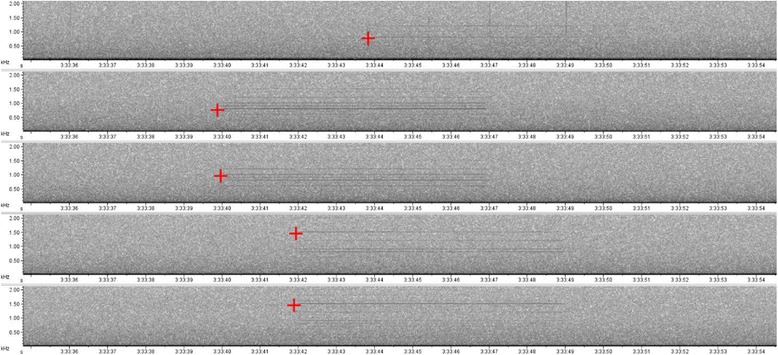
Time of arrival (TOA) measures based on a spectrogram obtained using Raven Pro software. Spectrogram view preset: Hann, 1024 samples, 90% overlap, time window length of 21.5 s, frequency range from 0 to 2000 Hz, greyscale color. Red crosses: pointers placed at the start positions detected in the broadcast sound signal

The two channels in all of the recordings were analyzed with Raven Pro software to detect the synthetic sound (version 1.5: [46]; Spectrogram view preset: Hann, 1024 samples, 90% overlap). We used the Sound Finder package in R software for localization estimation (see [47]). This free tool has a higher accuracy than other software [47]. To estimate the localization of a sound, the algorithm in this package requires the time of arrival (TOA) of sound to at least four microphones, the temperature (mean temperature in the study area during the night period), and the coordinates of the microphones. Among the two microphones on each recorder that recorded the sound (ideally four different recorders), we chose that with the best signal-to-noise ratio. When the sound was recorded by only three different recorders, the second microphone on the recorder with the best signal-to-noise ratio was used to obtain a total of four microphones. As the signal-to-noise ratio was too low to use cross-correlation or automatic detection algorithms, the TOA were measured manually based on the spectrogram view (Fig. 3). The TOA measures were repeated three times and then averaged.

Sound Finder was used to estimate the coordinates of the broadcast sites as well as the temporal error values. The temporal error is defined as the root-mean-squared error of the combined discrepancies between the theoretical and observed delays in the TOA for each pair of microphones [47]. It was used to evaluate the reliability of the localization estimates, where perfect localization had a temporal error of 0 ms.

The distance between the estimated localization (coordinates given by Sound Finder) and the actual broadcast site position (coordinates given by the GPS) corresponded to the localization accuracy. It was calculated using the distance matrix tool in QGIS software.

### Statistical analysis

All of the statistical analyses were conducted with R software (version 3.1.2: [48]) and results were considered to be statistically significant when *P* ≤ 0.05. All of the values were reported as the mean ± SD.

**Fig. 4:**
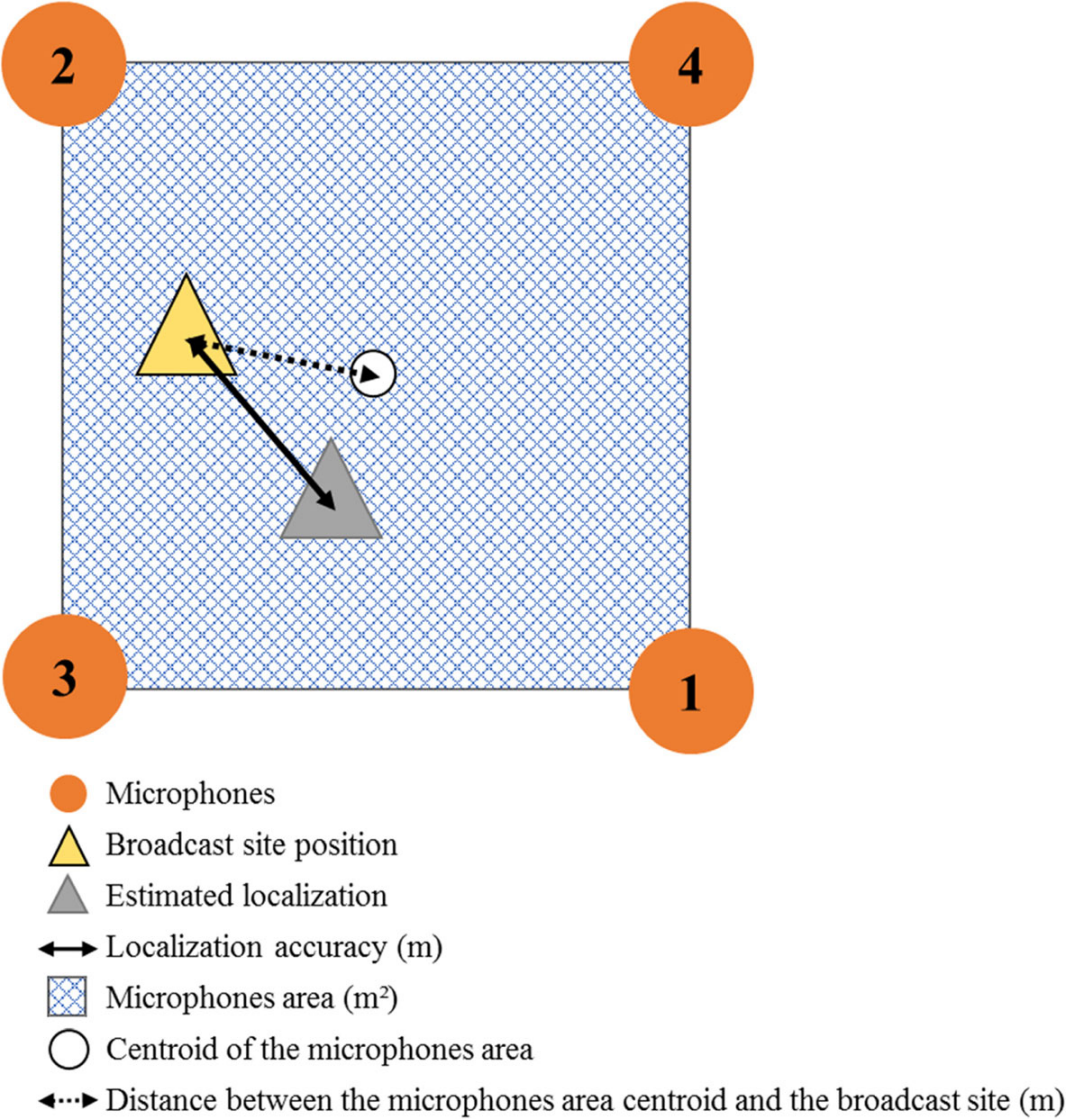
Representation of fixed effects when the broadcast site was positioned in the microphones area

Linear mixed-effects models (lmer function in the lme4 package: [49]) were used to identify parameters that influenced the localization accuracy (“*loc_accuracy*”). All combinations of the fixed effects and their interactions were used to construct the models. The four fixed effects (see Fig. 4) comprised the microphones area (“*areamic*” in m^2^), the distance between the microphones area centroid and the broadcast site (“*dist*” in m), the broadcast period (dusk, night, or dawn: “*period*”), and the broadcast site position compared with the microphones area (in or out: “*inout*”). Given our data structure, random effect was built as a nested random effect because the data came from two study areas (VM or CM: “*array*”) on three nights (N1, N2, or N3: “*night*”) and in three broadcast periods.

The best model was selected according to the lowest Akaike’s information criterion (AIC) [50]. The significance of fixed effects was tested using one-way analysis of variance (anova function in the MASS package: [51]) and random effect with the restricted likelihood ratio test (exactRLRT function in the RLRsim package: [52]).

## Results

### Broadcasts

In VM, two sites were excluded from the study because they were too dangerous to access during field nights. Thus, the synthetic sound was broadcast 174 times in VM and 180 times in CM with a total of 354 broadcasts. The broadcast sound amplitude remained constant during the three nights with a mean sound intensity level at 1 m of 115.04 ± 3.07 dBA in VM and 116.53 ± 3.59 dBA in CM. These values are close to the natural amplitude of wolf howls ([29], MP *unpublished observations*).

### Effectiveness of the recorders

All of the autonomous recorders were functional so the effectiveness of the experiments was 100%, with nearly 1200 h of acoustic recordings. According to visual and audio inspections of the recordings, 269 broadcasts were recorded by at least one autonomous recorder. In total, 101 broadcasts were recorded by one recorder (56 in VM and 45 in CM), 85 by two (36 in VM and 49 in CM), 55 by three (25 in VM and 30 in CM), 21 by four (three in VM and 18 in CM), and seven by five only in CM.

### TOA measures

The distances separating the broadcast sites and autonomous recorders ranged from 67 to 3595 m in CM and from 144 to 2751 m in VM. For several recordings, measures of the TOA were impossible to achieve because the signal-to-noise ratio was very low or the synthetic sound was only partially recorded and/or conspicuous. Thus, some recordings could not be included in the analysis. Finally, 59 broadcasts (17%), i.e. 14 in VM (8%) and 45 in CM (25%), recorded by at least four microphones were used for acoustic localization.

### Localization estimation

Localization estimates were calculated for the 59 broadcast sites (14 in VM and 45 in CM). The mean localization accuracy was about 315 ± 617 m and the mean temporal error was 685.57 ± 2049.73 ms (*N* = 59; Table 1). All of the usable broadcast sites in VM were located out of the microphones area whereas in CM, 28 were “out” and 17 were “in”. The mean distance between the microphones area centroid and the broadcast site was about 656.55 ± 422.09 m. The mean microphones area was 746,823 ± 342,362 m^2^ (N = 59).

**Table 1:** Localization accuracy and temporal error values estimated with Sound Finder. (a) With all localization estimations. (b) According to the 200 ms error reliability threshold

	Localization accuracy (m)			Temporal error (ms)			N
	Mean ± SD	Min	Max	Mean ± SD	Min	Max	
(a)							
VM	442 ± 597	28	1987	903.37 ± 2321.32	1.60	7865.69	14
CM	276 ± 625	1	2983	617.81 ± 1981.50	0.21	9389.91	45
Total	315 ± 617	1	2983	685.57 ± 2049.73	0.21	9389.91	59
(b)							
VM	316 ± 540	28	1987	29.67 ± 32.08	1.60	118.00	12
CM	123 ± 185	1	937	42.03 ± 53.29	0.21	189.84	41
Total	167 ± 308	1	1987	39.23 ± 49.29	0.21	198.84	53

**Fig. 5:**
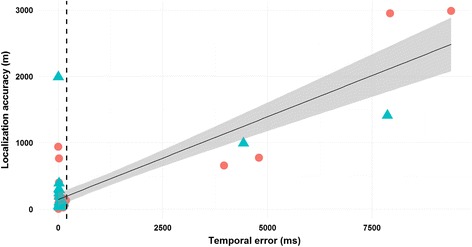
Relationship between the localization accuracy and temporal error in both study areas. Pearson’s correlation coefficient: *r* = 0.83, *P* < 0.001, *N* = 59. Vertical dotted line: 200 ms reliability threshold for the temporal error. Blue triangles: VM. Red circles: CM

There was a positive correlation between the localization accuracy and the temporal error value (Pearson’s correlation coefficient, *r* = 0.83, *P* < 0.001; Fig. 5), which indicated that the localization accuracy decreased when the temporal error increased. Based on this relationship, we identified a threshold in the temporal error above which the estimates were unreliable. After setting this reliability threshold to 200 ms, six inaccurate data (two in VM and four in CM) with high localization accuracy values (ranging between 534 and 3083 m) were excluded (see Fig. 5). The mean localization accuracy was then 167 ± 308 m (*N* = 53; Table 1). Considering the error threshold, all of the remaining data had accuracies less than 400 m, except three values having high localization accuracy and low temporal error values. These three aberrant data were excluded from the dataset used in the analysis of parameters influencing the localization accuracy.

### Parameters that influenced the localization accuracy

Eleven linear mixed-effects models were built in order to identify the parameters that influenced the localization accuracy (Table 2). Among the four fixed effects, only the broadcast site position relative to the microphones area (in or out) was not tested because both conditions were not present in VM (all sites positions were out). The mixed-model with the fixed effect *“dist”* had the lowest AIC (i.e. “m2”: AIC = 591.35). Thus, the distance between the microphones area centroid and the broadcast site significantly affected the localization accuracy (χ^2^ (1) = 11.27, *P* < 0.001). In particular, the localization accuracy was lower when the broadcast site was far from the microphones area centroid (estimate ± SE: 0.14 ± 0.04 m).

**Table 2:** Linear mixed-effects models used to identify parameters that influenced the localization accuracy. Models were built with fixed effects alone, summed (“+”) and with interactions (“*”). The nested random effect was similar for all of the models (see “m0”). Akaike’s information criterion (AIC) was calculated to select the best statistical model, i.e. the “m2” model

Models			
Name	Fixed effects	Random effect	AIC
m0	1	(1|array) + (1|array:night) + (1|array:night:period)	600.62
m1	period		604.35
m2	dist		591.35
m3	areamic		602.62
m4	period+dist		594.98
m4.int	period*dist		591.70
m5	period+areamic		606.31
m5.int	period*areamic		604.10
m6	dist+areamic		593.26
m6.int	dist*areamic		594.79
mc	period+dist+areamic		596.95

**Fig. 6:**
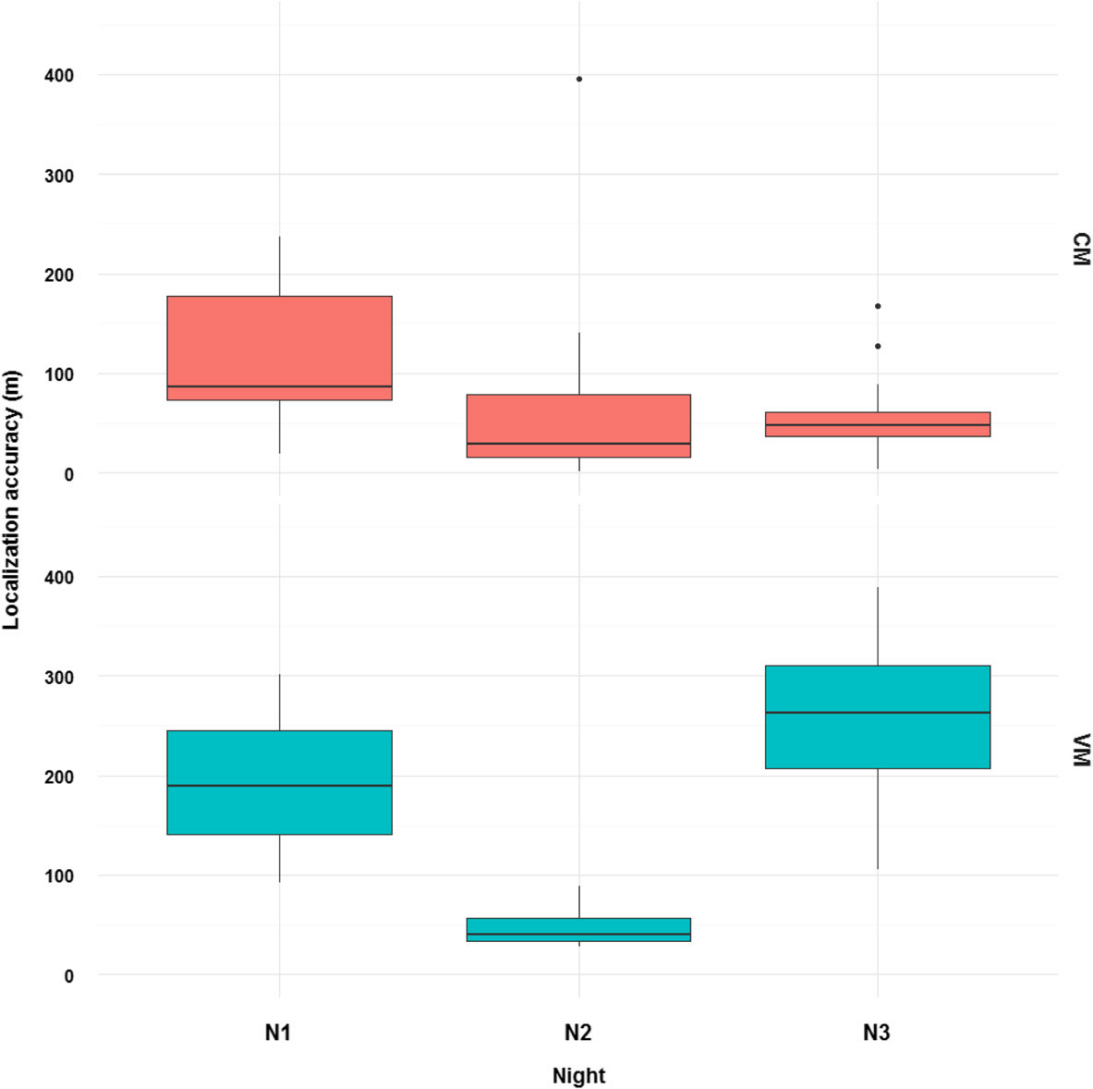
Variations in the localization accuracy on the three different nights in both study areas. Random effect of linear mixed-effect model: RLRT = 4.53, *P* < 0.05

Considering the random effect in the selected model, the localization accuracy did not vary between the two study areas (restricted likelihood ratio test [RLRT] = 0.06, *P* > 0.05) and there was no effect of period (RLRT = 0.05, *P >* 0.05). However, the localization accuracy varied significantly among the different nights inside each study area (RLRT = 4.53, *P* < 0.05; Fig. 6).

## Discussion

Since its natural return to France from the Italian population [53], the grey wolf first recolonized mountainous areas (French Alps) and its range is currently expanding west and northward into mid-mountain and lowland environments [19, 34–37]. Documenting and updating presence and localization of wolves is crucially important for managing this protected species and for preempting potential conflicts with human activities, especially livestock attacks. Thus, in this study, we investigated a new, non-invasive, and large-scale acoustic method for localizing wolves.

### Acoustic localization estimates

In our study, 76% of the broadcasts were recorded by at least one recorder, thereby demonstrating the potential for using a low-density microphone array to detect howls over large areas (30 km^2^) with contrasting environmental contexts. The 59 broadcasts recorded by at least four microphones were used to estimate localizations. Although accuracies did not differ significantly between the two study areas, we observed a difference in the sample size of the broadcasts recorded and used for localization estimation, particularly in the lowland environment (45 in CM) compared with the mid-mountain environment (14 in VM).

After considering the relationship between the localization accuracy and the temporal error value, we defined a reliability threshold for the temporal error. We set this threshold to 200 ms. Then, most of the inaccurate values were excluded conducing to a mean localization accuracy of less than 200 m. This value may be considered a poor localization estimate when compared with most studies of acoustic localization [2, 47, 54, 55]. However, these previous studies were conducted in much smaller study areas. Thus, considering the distance between the autonomous recorders in our experiments, a localization accuracy of 200 m appears to be consistent.

In addition, we showed that some parameters could influence the localization accuracy and so, should be considered to optimize future protocols. First, the localization accuracy varied among different nights in each study area and at the same broadcast site (replicate). The wind speed was negligible during the experiments, but variations in other meteorological conditions among different nights may explain the differences in accuracy (e.g. air temperature or wind direction). Indeed, the meteorological conditions are known to have strong effects on sound propagation and signal detection, and thus on the localization accuracy [56]. We also showed that the distance between the microphones area centroid and the broadcast site had a significant effect on the localization accuracy. The localization accuracy was lower when the broadcast site was far from the microphones area centroid, as shown in previous studies [2, 54, 57].

### Recommendations and perspectives for grey wolf monitoring

According to our results, some recommendations may be made regarding the development of effective acoustic methods for grey wolf monitoring. The measures of the TOA were performed manually and this was a time-consuming task. Automatic and autonomous methods for detecting wolf howls in recordings and then for localizing them, such as methods based on temporal cross-correlation, could improve the results and save time. However, these methods are still very complicated [58, 59]. Moreover, amplitude and frequency modulations in wolf howls may make difficult to parameterize a unique automatic detector that could be trusted without human verification.

As shown in the present study and previous investigations (e.g. [2, 54, 57]), the distance between the sound source and the microphones area centroid influenced the localization accuracy. Similarly, during field recordings, large distances between the study species and recorders may also influence the localization accuracy because of a low signal-to-noise ratio (as found in our study). Thus, the selection of the recording sites should be optimized according to the ecology and behavior of wolves but also based on local expert knowledge in order to increase the likelihood of collecting acoustic data.

The structure and the composition of the landscape, such as the topography and vegetation (e.g. composition and stand density), could also influence the localization estimations, and thus they should be considered when defining protocols based on acoustic methods. This may partly explain the difference in the sample sizes for the broadcasts used in the lowland and mid-mountain environments. Previous studies also demonstrated that the optimal placement of recorders is important for ensuring maximum cover of the study area [29, 54].

These recommendations highlight the necessity to find a compromise between the distance that separates the microphones, the area covered by the microphone array, the areas where vocalizations or sounds are produced, and the desired localization accuracy [54]. Considering our results and soundscape parameters, it would be interesting to model the sound detection space of the autonomous recorders in order to place them optimally in the field and to improve the localization accuracy.

Finally, the acoustic localization protocol may concern much more wolves living in pack rather than dispersers or lone wolves (less frequent howls; [20]). This potential limit could be balanced by combining autonomous recorders with howling playback method to elicit wolves to howl [60]. This would be even more recommended in the colonization fronts (like in north-eastern France) for monitoring wolf dispersion but also for detecting new pack installation [61].

## Conclusions

Currently, monitoring of the distribution and demographic dynamics of the grey wolf in France is based on the standardized collection of presence signs by a network of 3500 trained volunteers [62]. Different methods are used such as opportunistic survey (scat, hair, saliva, etc.), non-invasive genetics analysis, intensive snow-tracking during the winter, and wolf howling in summer to detect breeding events [62]. However, the potential use of acoustic and autonomous recorders has not been considered for localizing individuals as well as specific areas such as rendezvous sites in contrasting environments. Thus, the development of a localization protocol based on passive acoustic methods could help scientists and decision-makers to collect new data to understand and monitor wolf recolonization. Importantly, these data could help prevent or mitigate conflicts with human activities as well as being used for conservation and management purposes.

Today, more than ever, large scale studies for monitoring elusive species are necessary and remain challenging [63]. Localization protocols based on our results and recommendations could be applied to species producing long-distance acoustic signals, even in large territories and contrasting environments. This kind of protocols will considerably help to monitor the conservation status of many elusive species in the long term.
